# Myeloid-derived Suppressor Cells and Multiple Sclerosis

**DOI:** 10.2174/1570159X22999240710142942

**Published:** 2024-07-10

**Authors:** Aurora Zanghì, Paola Sofia Di Filippo, Carlo Avolio, Emanuele D’Amico

**Affiliations:** 1 Department of Medical and Surgical Sciences, University of Foggia, Foggia, Italy

**Keywords:** Multiple sclerosis, myeloid-derived suppressor cells, M-MDSCs, G-MDSCs, therapeutic strategies, immune responses

## Abstract

Myeloid-Derived Suppressor Cells (MDSCs) are a heterogeneous population of immature myeloid cells that play important roles in maintaining immune homeostasis and regulating immune responses. MDSCs can be divided into two main subsets based on their surface markers and functional properties: granulocytic MDSCs (G-MDSCs) and monocytic MDSCs (M-MDSCs). Recently greatest attention has been paid to innate immunity in Multiple Sclerosis (MS), so the aim of our review is to provide an overview of the main characteristics of MDSCs in MS and its preclinical model by discussing the most recent data available. The immunosuppressive functions of MDSCs can be dysregulated in MS, leading to an exacerbation of the autoimmune response and disease progression. Antigen-specific peptide immunotherapy, which aims to restore tolerance while avoiding the use of non-specific immunosuppressive drugs, is a promising approach for autoimmune diseases, but the cellular mechanisms behind successful therapy remain poorly understood. Therefore, targeting MDSCs could be a promising therapeutic approach for MS. Various strategies for modulating MDSCs have been investigated, including the use of pharmacological agents, biological agents, and adoptive transfer of exogenous MDSCs. However, it remained unclear whether MDSCs display any therapeutic potential in MS and how this therapy could modulate different aspects of the disease. Collectively, all the described studies revealed a pivotal role for MDSCs in the regulation of MS.

## 1 INTRODUCTION

Myeloid-derived suppressor cells (MDSCs) are a heterogeneous population of immature myeloid cells that play important roles in maintaining immune homeostasis and regulating immune responses [1]. MDSCs were first identified in cancer patients as a population of cells that accumulate in the blood, lymphoid organs, and tumor microenvironment [1, 2]. Later, MDSCs were also found to play a role in other pathological conditions, including chronic infections and autoimmune diseases [3, 4].

MDSCs are generated from bone marrow-derived myeloid progenitor cells under conditions of inflammation and immune activation. The differentiation of MDSCs is regulated by various factors, including cytokines, chemokines, and growth factors, which are produced in response to inflammation and infection [1]. These factors induce the expansion and differentiation of myeloid progenitor cells into MDSCs, which are then released into the circulation and migrate to sites of inflammation and immune activation [5].

MDSCs can be divided into two main subsets based on their surface markers and functional properties: granulocytic MDSCs (G-MDSCs) and monocytic MDSCs (M-MDSCs).

In mice, M-MDSCs are defined by the surface phenotype CD11b+Ly6G−Ly6Chi and G-MDSCs by CD11b+ Ly6G+Ly6Clo [6]. In humans, M-MDSCs are CD14+HLA-DR−/lo and G-MDSCs CD11b+CD15+CD14-CD33+/ loCD66b+ cells with a low density [7-9] and are present in the peripheral blood mononuclear fraction of gradient centrifugations. Many markers are still appearing in efforts to further define the human MDSC subsets [10], one being S100A9 [11, 12]. G-MDSCs can differentiate into granulocytes, such as neutrophils, basophils, and eosinophils. These cells are known to accumulate in various pathological conditions, including cancer, inflammation, infection, and autoimmune diseases [13-15].

The primary function of G-MDSCs is to suppress the activity of T cells and other immune cells, which is believed to be a mechanism that helps the body avoid excessive inflammation and tissue damage [8, 15].

Substantial evidence now shows that monocytes and macrophages are prominent myeloid cell types during early disease stages and mediate both pro- and anti-inflammatory responses [16-19].

Furthermore, M-MDSCs, under specific conditions, may differentiate into dendritic cells (DCs), contributing to the overall immune regulatory functions of MDSCs [20].

MDSCs immunosuppressive activities are mediated by multiple mechanisms. These mechanisms include the depletion of essential amino acids such as arginine and cysteine, production of reactive oxygen species (ROS), production of nitric oxide (NO), and expression of inhibitory molecules such as programmed death-ligand 1 (PD-L1) and cytotoxic T lymphocyte-associated antigen 4 (CTLA-4). These mechanisms lead to the inhibition of T-cell activation, proliferation, and effector functions [21-24].

Moreover, MDSCs can inhibit antigen presentation by downregulating the expression of major histocompatibility complex (MHC) class II molecules on antigen-presenting cells (APCs) and decreasing the expression of costimulatory molecules such as CD80 and CD86 [3].

Understanding the biology of MDSCs has been an active area of research in the immunology field. Therapeutic approaches targeting MDSCs are being explored to potentially modulate their activity and improve immune responses in individuals with dysregulated immune function.

Specifically, the role of MDSCs has been deeply investigated in the central nervous system (CNS) demyelinating diseases such as Multiple Sclerosis (MS). MS is a chronic, progressive disease that leads to increasing disability in many individuals [25, 26]. The symptoms of MS can range from mild to severe and may include fatigue, muscle weakness, numbness or tingling, vision problems, difficulty with coordination and balance, and cognitive impairment [25, 27]. It is a highly variable disease, and individuals may experience different disease courses and levels of disability. Approximately 85 percent of individuals initially present with a relapsing-remitting (RRMS) course of the disease [28]. Most people with RRMS experience a transition to a more progressive course, secondary-progressive MS (SPMS) that is characterized by the accumulation of disability with fewer relapses [28]. About 10 to 15 percent of people with MS begin the disease with a progressive course, without relapses, known as primary progressive MS (PPMS) [28]. Currently, the course of the disease is regarded as a continuum, thus distinguishing the presence of activity and progression as elements that can be present from disease onset [26, 29].

The fundamental pathology of MS is characterized by “inflammatory demyelination,” and various efforts have been made to investigate the underlying cause of this inflammation [30-32]. Conversely, the endorsement of its most commonly used model, experimental autoimmune encephalomyelitis (EAE), has substantiated the hypothesis that the initiation of MS is associated with “inflammatory demyelination” resulting from an autoimmune mechanism [33].

While the complete pathogenesis of MS remains unknown, several presumed mechanisms have been identified. Activated CD4+ T helper type 1 (Th1) cells, stimulated by peripheral antigen presentation, traverse the blood-brain barrier (BBB) and infiltrate the CNS [33, 34]. Within the CNS tissue, Th1 cells, represented by antigen-presenting cells, undergo reactivation and produce Th1 cytokines [32]. Th1 cytokines, in turn, stimulate the differentiation of B cells, leading to the production of antibodies against self-antigens and triggering an antigen-antibody reaction in the myelin sheath [32]. Additionally, cytotoxic macrophages, activated by Th1 cytokines, generate substantial amounts of inflammatory cytokines, contributing to demyelination alongside antibodies [32].

While the exact role of the innate immune system and its association with different stages of MS remains unclear, it is postulated that components of the innate immune system contribute to various detrimental steps in the autoimmune cascade. This includes the activation of myelin-reactive T lymphocytes by APCs and the formation of membrane attack complexes in the CNS [35]. In MS patients, inflammatory lesions have been identified within the CNS, surrounded by infiltrating T lymphocytes, monocytes, and macrophages, as well as activated microglia and reactive astrocytes [36]. This observation suggests a pivotal role of the innate immune system in mediating neuronal damage [36]. There is broad heterogeneity in how the disease-modifying therapies (DMTs) for MS are used in clinical practice, mainly because of the multitude of available treatment options [37]. In addition, recent international consensus guidelines offer differing recommendations regarding the approach to DMTs: historically, an escalation approach was used for newly diagnosed patients with RRMS, but the evidence for clinical benefits of early treatment with high-efficacy therapies (HETs) in this population is emerging [37].

The aim of our review is to provide an overview of the main characteristics of MDSCs in MS and its preclinical models by discussing the most recent data available, so we sought to define the possible therapeutic targets of MDSCS in the landscape of MS with the intent to define the clinical and pathogenic associations reported to date in the literature.

## 2 METHODS

We employed the PubMed Search engine to perform a bibliographic search of the relevant literature available on the MEDLINE electronic database as described in appendices **1A** and **1B**. This resulted in 34 articles, which were subsequently reviewed based on their titles, abstracts and full manuscripts. The Relevant articles were assessed, and their references were reviewed to identify pertinent articles that did not result from the initial query. Only articles pertaining to the MDSC/MS disease model and MS were ultimately included in the present study.

## 3 MDSCS IMMUNOMODULATORY FUNCTIONS: AN OVERVIEW

**Fig. (1):**
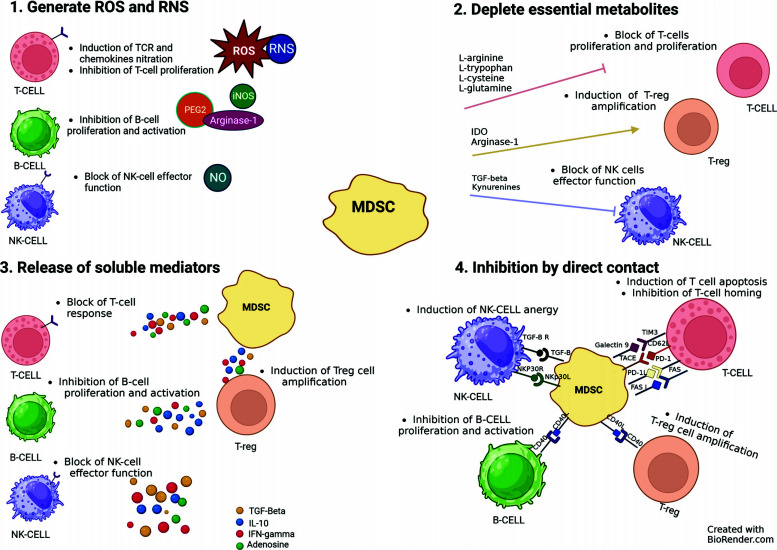
MDSC immunomodulatory functions. (**1**) MDSCs generate ROS and RNS. (**2**) MDSCs deplete essential metabolites (**3**) MDSCs release soluble mediators. (**4**) MDSCs inhibit immune cells by direct contact.

In healthy individuals, MDSCs contribute to immune surveillance and immune tolerance through several mechanisms: (1) MDSCs generate ROS and reactive nitrogen species (RNS). (2) MDSCs deplete essential metabolites. (3) MDSCs release soluble mediators. (4) MDSCs inhibit immune cells by direct contact (Fig. **1**).

### 3.1 MDSCs Generate ROS and RNS

The main source of ROS in MDSCs is nicotinamide adenine dinucleotide phosphate oxidase (NADPH), which produces superoxide radicals by transferring electrons from NADPH to oxygen [38]. The accumulation of ROS reduces cytokine secretion from T cells [38]. Additionally, the release of RNS inhibits the recruitment and proliferation of T cells by nitration/nitrosylation of T-cell receptor (TCR) and chemokines [38]. MDSCs directly repress the proliferation and activation of B cells through various mechanisms, including prostaglandin E2 (PGE2), inducible nitric oxide synthase (iNOS), and Arg-1 [38]. Moreover, MDSCs suppress Natural Killer (NK) cell immune functions by releasing nitric oxide (NO) to inhibit the FC-receptor-mediated antibody-dependent cell-mediated cytotoxicity (ADCC) [38].

### 3.2 MDSCs Deplete Essential Metabolites

MDSCs deplete amino acids needed for the metabolism of T cells, such as L-arginine. Promoted by the highly active Arg-1 secreted by MDSCs, L-arginine depletion causes a reduction in T cell stagnation, thereby altering the immune response of T cells [39].

Additionally, MDSCs, through the synergistic effect of indoleamine 2,3-dioxygenase (IDO) (a rate-limiting enzyme in tryptophan metabolism), promote the differentiation of regulatory T (Treg) cells and indirectly inhibit the immune response. IDO also catabolizes kynurenine and inhibits NK cell activation and proliferation [39].

### 3.3 MDSCs Release Soluble Mediators

MDSCs release soluble mediators such as adenosine that impact NK cells maturation but can also affect the effector functions of NK and T cells [40]. Additionally, by releasing IFN-γ, TGF-β, and IL-10, MDSCs restrict the proliferation and cytotoxicity of T cells and can promote the amplification of Treg cells [40].

### 3.4 MDSCs Exert the Inhibition of Immune Cells Through Direct Contact

MDSCs induce the apoptosis of T cells by expressing Galectin 9, Programmed Death-Ligand 1 (PD-L1), and Fas Ligand (FAS-L), which binds to corresponding receptors on the surface of T cells [41]. Moreover, MDSCs hamper naïve T cells homing through CD62L-TNF-α Converting Enzyme (TACE) interaction and promote NK cells anergy through TGF-β-NKp30L interaction. MDSCs induce the amplification of Treg cells and inhibit the proliferation of B cells through CD40-CD40L interactions [42].

Gaining a comprehensive understanding of these typical functions of MDSCs offers valuable insights into their possible implications in various diseases and guides the formulation of therapeutic approaches aimed at regulating their activity in pathological states.

## 4 MS DISEASE MODELS AND MDSCs

The therapeutic and protective effects of MDSCs and the role of pivotal cytokines on immunomodulatory cells have been investigated on the most employed MS disease models, EAE. Another MS disease model for the progressive form that has also been investigated is Theiler’s virus-induced demyelinating disease (TMEV-IDD) (Table **1**).

In an Iranian study, mice were immunized to develop the EAE model and divided into different groups that underwent pre-EAE induction treatment or post-EAE induction treatment with Tregs, G-MDSCs or IL-2 [43]. Pre-treatment with G-MDSCs prevented EAE induction, while post-treatment with G-MDSCs mediated EAE remission, suggesting that these cells strongly repress the clinical and pathological properties of EAE and may be used to develop cell-based therapies in patients with autoimmune inflammatory diseases [43].

In a study by Melero-Jerez *et al*. [44], the abundance of M-MDSCs correlates with the density of oligodendrocyte precursor cells (OPCs) in EAE demyelinating lesions, suggesting that M-MDSCs might also have a direct influence on OPCs and remyelination.

Here, the authors describe the effects of M-MDSCs as promoters of OPC differentiation and for the first time, osteopontin was supposed as the main M-MDSCs effector on these oligodendrial cells [44].

These data highlight a crucial pathogenic interaction between innate immunity and the CNS, opening ways to develop M-MDSCs- and/or osteopontin-based therapies to promote effective myelin preservation and repair in MS patients [44].

Another study showed a direct correlation between the splenic M-MDSCs population and the preservation of myelin and axons, which was also correlated with T cell apoptosis within the CNS (being these cells the main target for M-MDSCs suppression) [45]. The data suggested a clear relationship with disease outcome, opening new perspectives for the future targeting of this population as an indicator of MS severity [45].

Furthermore, also anti-inflammatory properties of cannabidiol (CBD), a non-psychoactive cannabinoid (CB), have been investigated [46]. Interestingly, CBD treatment led to a profound increase of G-MDSCs cells in EAE mice when compared to the vehicle-treated EAE controls and these cells ameliorated EAE [46]; in the counterpart, this effect was reversed after G-MDSCs depletion [46]. Thereby, the authors suggested that anti-inflammatory CBD functions were mediated by the induction of immunosuppressive G-MDSCs [46]. Mecha *et al*. [47] used a TMEV-IDD as a model of acute neuroinflammation to investigate the role of 2- arachidonoylglycerol (2-AG), the implication of CB1 and CB2 receptors in 2-AG actions and the modulation of immune cells trafficking into the CNS. Here, the 2-AG or the inhibition of its hydrolysis increased the number and activity of M-MDSCs within the infected brain and diminished the reactivity and number of microglia at the TMEV injection site, reducing their morphological complexity and modulating them towards an anti-inflammatory state *via* CB2 receptors, highlighting the interest of modulating endogenous CBs to regulate CNS inflammatory conditions [47].

A more comprehensive description of the role of M and G-MDSCs in a TMEV-IDD model can be found in Bowen *et al*.’s work [48]. This study demonstrated that the foremost infiltrating immune cells during the innate immune response to TMEV are M-MDSCs, while G-MDSCs showed very limited infiltration properties of the infected brain, which were nearly absent the second day after the infection [48]. In contrast with previously presented findings, depletion of M-MDSCs in this model reduced the development and progression of demyelinating disease due to an increase in CD4+ T cell and CD8+ T cell responses against the virus, which enabled a more effective virus clearance [48].

The various subtypes of MDSCs have also been examined within the context of EAE to elucidate the induction of immune system tolerance.

A study by Wegner *et al*. [49] revealed an involvement of G-MDSCs in the induction of immune system tolerance through inhibition of CD4+T cells. G-MDSCs were shown to adopt a more suppressive phenotype during peptide immunotherapy and inhibit CD4+ T-cell proliferation in a cell-contact-dependent manner mediated by Arg-1 [49]. Moreover, increased numbers of tolerogenic G-MDSCs, as observed during peptide immunotherapy, were demonstrated to protect from disease in an experimental model of EAE [49].

A study by Casacuberta *et al*. [50] showed that M and G-MDSCs expressing a self-antigen can induce apoptosis of CD4+ T cells *in vitro*, thus mediating specific immune tolerance in a murine model of autoimmunity.

Ioannou *et al*. [51] built a translational model and evaluated the exposure of G-MDSCs to the autoimmune milieu led to up-regulation of the programmed death 1 ligand that was required for the G-MDSCs-mediated suppressive function both *in vitro* and *in vivo*. Importantly, MDSCs were enriched in the periphery of subjects with active MS and suppressed the activation and proliferation of autologous CD4(+) T cells *ex vivo* [51]. In a pre-clinical model, *in vivo* transfer of highly purified G-MDSCs ameliorated EAE [51]. Importantly, these results demonstrated, for the first time, an important role for G-MDSCs in patients with MS because this subset was significantly increased in the periphery during active disease and potently suppressed autologous T cell proliferation *in vitro*. Together, these data highlight the potential of G-MDSCs to serve as a novel target for pharmacologic intervention in autoimmune inflammatory diseases [51].

Through the study of oxidized mannan-conjugated myelin oligodendrocyte glycoprotein (MOG) 35-55, another research study showed that peripheral maturation of M-MDSCs monocytes to PD-L1+ cells is sufficient to reverse spinal cord inflammation and demyelination in MOG-induced EAE [52]. Then, this monocyte maturation by oligodendrocyte-myelin peptides represents a novel mechanism of immune tolerance that reverses EAE [52]. The importance of PD-L1/PD-1 mediated interactions between M/G-MDSCs and T cells was also demonstrated in a study by Ishihara *et al*. [53] in which, upon treatment of EAE mice with serum albumin (SA)-IL-4 fusion protein, PD-L1 and PD-1 expression increased respectively on both subsets of MDSCs and T cells. This produced a reduction of immune infiltration into the spinal cord of the animals, thus preventing EAE development in the majority of the tested mice [53].

A research investigation focusing on the MDSCs population in the spinal cord during EAE revealed the presence of Arg-I(+)-M-MDSCs cells, which were primarily localized within the demyelinating plaques [54]. The prevalence and concentration of Arg-I+ cells, coupled with the ratio of apoptotic rather than proliferative T cells, exhibited a congruence with the temporal advancement of EAE, achieving a zenith concurrent with the clinical score, manifesting a notable reduction during the remission stage and ultimately vanishing during the chronic phase [54]. Upon extraction of M-MDSCs from the spinal cord of EAE animals and their subsequent co-culture with stimulated control splenic CD3 T cells, they elicited an enhancement in cell death [54]. These findings underscore the noteworthy role exerted by MDSCs in attenuating inflammatory harm in MS. Consequently, the MDSC population emerges as a potential therapeutic target to expedite the recovery of MS patients [54]. Accordingly, Wang *et al*. [55] demonstrated that treatment of EAE mice with the potentially anti-inflammatory NAD+ increased splenic levels of Arg-I(+)-G-MDSCs while diminishing clinical scores of EAE and slightly delaying disease onset.

The positive activities of M-MDSCs have also been investigated in two studies by Zhu *et al*. [56, 57]. They revealed that M-MDSCs increase in the spleen after EAE induction and suppress CD4 T cell proliferation *in vitro* [56]. Then, *in vivo* transfer of activated M-MDSC enhances T cell apoptosis in the CNS and markedly suppresses EAE, delaying the onset and reducing the incidence and severity of the disease [57].

Beneficial effects of M-MDSCs were unveiled as well in a work by Ortega *et al*. [58]. Here, it was demonstrated that higher levels of M-MDSCs in the peripheral blood of EAE mice at disease onset correlate with the future milder disease severity [58]. At the same time, peripheral M-MDSCs exerted strong immunosuppressive activity over both CD4+ and CD8+ MOG-stimulated T cells *in vitro* [58].

The pleiotropic effect of MDSCs on T cells and their ability to influence organ-specific targets such as the CNS from remote organs could elucidate mechanisms of the disease and could provide new therapeutic targets.

For this purpose, a study by Glenn *et al.* [59] discussed on remote activation of Th17 cells by lung myeloid cells during EAE. Their results indicate a robust accumulation of G-MDSCs in the lungs of mice during EAE, which could promote Th17 polarization and coincide with the trafficking of autoimmune-targeted T cells through the lungs [59, 60].

The results of a study by Yi-H *et al*. [61] unveil a complicated inflammatory network in autoimmune disorders such as EAE. The authors discovered that mouse G-MDSCs can drive the differentiation of Th17 cells under Th17-polarizing conditions (*e.g*., in the presence of cytokines IL-6 and TGF-β) [61]. Instead of curbing T cell activation or facilitating the resolution of inflammation, the excessive expansion and the prolonged accumulation of G-MDSCs may have detrimental effects by enhancing the development of Th17 cells, which can further drive tissue inflammation and aggravate tissue damage in autoimmune diseases [61].

Another study provides new insights into the host-microbiota interactions in EAE, suggesting that activated M-MDSCs could be potentially used as an efficient therapy for acute phases of MS [62].

Here, M-MDSCs differentiated with prostaglandin PGE2 and control M-MDSCs (differentiated without PGE2) reduced infiltration of Th17 and IFN-γ-producing NK cells and an increased proportion of Treg cells in the CNS and spleen [62]. Importantly, only M-MDSC-PGE2 prevented the extensive alterations in gut microbiota composition due to their early migration into Payer's patches and mesenteric lymph nodes [62]. Gut microbial taxa were significantly enriched, with taxa having immunoregulatory properties [62].

The MDSCs have also been studied for their interactions with invariant NKT cells [63]. Here, the authors provided strong evidence for cooperation between invariant NKT cells with M and G-MDSCs in protection against autoimmunity in the CNS conferred by the invariant NKT cell agonist α-GalCer [63]. Undoubtedly, that might be exploited for the development of improved immunotherapies for MS and other autoimmune and inflammatory diseases [63].

Additionally, a study by Alabanza *et al*. [64] showed that the inhibition of endogenous activated protein C affected EAE pathogenesis at multiple fronts [64]; specifically, activated protein C inhibition modulated the functional responses of CD11b+ cells, leading to the expansion and increased activation of both M- and G-MDSCs, (suppressive to the CD4+ T-cells required for EAE progression) thereby resulting in attenuated EAE [64].

Another study by Moliné-Velázquez *et al*. [65] specifically focused on the synthetic retinoid acid analogue retinoid Am80. Am80 is a novel RARα/β-specific synthetic retinoid that shows approximately 10-fold greater effective biological activity than all-trans-retinoic acid [65]. In this work, the authors aimed to determine whether MDSCs differentiation may affect the clinical course of EAE by hindering or delaying the transition from the relapsing period to the partial recovery [65]. The reported data indicated that Am80 induces M-MDSCs apoptosis and therefore, increases T cell viability and severe effects of EAE [65]. Am80 also induces M-MDSCs polarization to dendritic cells, macrophage or neutrophil phenotype, favouring their pro- rather than their anti-inflammatory properties [65]. These data suggested that undifferentiated MDSCs are crucial cells that must be taken into consideration when designing future disease-modifying treatments for MS [65].

Regarding the negative aspects of MDSCs, a study by King *et al*. [66] unveiled a potential pathogenic role of circulating M-MDSCs during demyelinating autoimmune disease. In this work, an expansion of M-MDSCs in the blood of mice before and during EAE exacerbations was found [66]. Additionally, these circulating cells were found to be the source of mature and CNS-infiltrating myeloid cells, which showed a pro-inflammatory phenotype characterized by up-regulation of MHC class II and increased production of pro-inflammatory cytokines, like IL-23 and IL-6 [66]. Another evidence of the detrimental effects of M-MDSCs in demyelinating diseases comes from Zhang *et al*.’s work [67]. Here, treatment with Pseudolycorine chloride, an alkaloid extracted from Narcissus tazetta with anti-tumor and anti-viral properties, alleviated the EAE course by reducing M-MDSCs expansion in the peripheral blood of EAE mice [67]. This prevented M-MDSCs-dependent differentiation of Th17 cells within the spinal cord of affected mice, thus reducing inflammatory infiltration and demyelination [67].

These conflicting results could potentially come from high levels of plasticity of MDSCs during the fast immunological changes in EAE: an important factor that should be taken into consideration when performing M-MDSCs' analysis during EAE is the timing in order to gain a better understanding of when and how MDSCs-driven immunomodulation occurs [68].

Another investigated setting with increasing resonance is the role of age and immune response. Hertzenberg *et al*. [69] demonstrated that the induction of EAE using a standard protocol is unfeasible in mice susceptible to the disease but below a specific age threshold. Disease resistance observed in the younger mice correlated with an elevated frequency of plasmacytoid DCs and M-MDSC (both APC subtypes possessing immunosuppressive attributes) [21].

Moreover, APC derived from the younger mice exhibited a functionally immature phenotype, characterized by diminished expression of MHC II and co-stimulatory molecule CD40 [69]. These APCs also exhibited reduced production of pro-inflammatory cytokines such as TNF, IL-6, IL-23, and IL-12, alongside heightened release of the anti-inflammatory cytokine IL-10 [69]. Furthermore, these APCs demonstrated an inability to generate encephalitogenic T cells, instead fostering the development of regulatory T cell populations [69]. These findings proposed a decline in regulatory APC phenotypes and M-MDSCs associated with age, alongside an augmentation in the expression of constitutive and inducible MHC II and co-stimulatory molecules on myeloid APCs and B cells [69].

This age-related shift in immune profile explains the protective effect observed in young mice against T cell-mediated CNS autoimmune diseases.

The impact of DMTs in MS disease models has been explored in a limited number of studies.

One of them showed that a single injection of interferon-beta (IFN-β) at the onset of the clinical course reduces the severity of the EAE, enhancing the presence of M-MDSCs within the smaller demyelinated areas, promoting M-MDSCs immunosuppressive activity both *in vivo* and *in vitro*, augmenting T cell apoptosis [70]. Additionally, IFN-ß preserved the immature state of M-MDSCs, impeding their progression toward mature and less suppressive subtypes of myeloid cells [70]. All these components offer novel insights into the potential status of M-MDSCs as an inherent mediator of their advantageous function within this animal model of MS [70]. Another study elucidated that the signalling mediated by type I IFNs within Tregs attenuates their capacity to curb chemokine production [71]. These elements brought a consequential alteration of the overall microenvironment within the draining lymph nodes, thereby fostering the recruitment of MDSCs and yielding beneficial effects on the disease outcome [71].

Another study by van der Touw *et al*. [72] demonstrated that glatiramer acetate (GA) interacts with murine-paired Ig-like receptor B on MDSCs and suppresses the STAT1/NF-κB pathways while promoting IL-10/TGF-β cytokine release. Analyses of cytokine profiles also indicated that MDSCs and GA both contributed to the reduction of IL-17 [72]. Then, T-reg-promoting properties of GA result, in part, from altered cytokine release from innate immune cells [72].

Furthermore, a translational study on G-MDSCs in cerebrospinal fluid (CSF) showed that loss of MDSCs leads to activation of B cells in the CNS, which – in part by secretion of Granulocyte-Macrophage Colony-Stimulating Factor (GM-CSF) contribute to the establishment of compartmentalized inflammation in the CNS [73]. It was supposed that B cells could potentially induce a phenotype resembling MDSCs in a specific subset of neutrophils within the CNS [73]. This interaction could establish a negative feedback loop aimed at inhibiting the sustained propagation of inflammation within the CNS [73].

These results suggested that MDSCs in the CNS could prevent the accumulation of B cells in the meningeal space and CNS parenchyma [73]. Upon relief from MDSCs-mediated suppression, B cells accumulate in the CNS compartment, even in cases of MOG-induced EAE [73]. Through their secretion of inflammatory cytokines, particularly GM-CSF, these B cells contribute to the propagation of immunopathology [73].

In conclusion, the authors suggested an interaction between G-MDSCs and a subset of IL-6 and GM-CSF-producing B cells in CNS autoimmunity [73]. These two immune cell subsets are linked in a negative feedback loop [73]. Therapeutic approaches targeting the manipulation of this interaction could potentially prevent the continued propagation of inflammatory responses within the CNS compartment in chronic autoimmune diseases, particularly when localized clusters of B cells drive immunopathology [73].

Recently, in a translational study, M-MDSCs were quantified in MS patients at baseline and correlated with different clinical parameters after 12 months of fingolimod treatment [74]. M-MDSCs at baseline were highly representative of a good therapeutic response to fingolimod (patients who met at least two of the criteria used to define non-evidence of disease activity 3, 12 months after treatment [74]. These data displayed a clear parallel with the preclinical results reported previously in the EAE mice where M-MDSCs abundance was used to discriminate a good response to fingolimod [74]. Moreover, M-MDSCs were enriched in patients whose disability remained stable or diminished after 12 months of fingolimod [74].

## 5 MULTIPLE SCLEROSIS AND MDSC

The role of MDSCs has been examined in a restricted context of MS, utilizing cytofluorometric analyses as a common approach (Table **2**).

A real-world Italian case-control study aimed to also characterize the myeloid cells in addition to B, and T cells in patients recently diagnosed with RRMS naive to DMTs [75]. Myeloid cell analysis showed that patients had higher M-MDSCs CD14+/HLADR-/low (9.3%, SD 3.2% *vs.* 7.5%, SD 1.8%, *p* = .022) and inflammatory monocytes, CD14+/ CD16+ (12.5%, SD 5.6% *vs.* 5.6%, SD 2.8%, *p* = .002) when compared with healthy controls (HCs) [75]. The discriminatory power through receiving operator curves of M-MDSCs CD14+/HLADR−/low and inflammatory monocytes, CD14+ CD16+, populations was >70% (area under the curves); thus, these cell subsets were supposed to be a disease biomarker [75]. In this context, the authors hypothesized that the inflammatory monocyte CD14+CD16+ counterparts could potentially serve as disease biomarkers in comparison to HCs.

Iacobaeus *et al*. [76] assessed the frequencies of CD14+ HLA-DRlow M-MDSCs and CD33+CD15+CD11b+HLA-DRlow G-MDSCs and investigated phenotypic and functional differences of M-MDSCs in different disease phases: RRMS patients during disease activity, RRMS patients stable and secondary progressive patients. They were also compared to HCs [76]. Increased frequencies of M-MDSCs (*p* < 0.05) and G-MDSCs (*p* < 0.05) were observed in RRMS patients during relapse compared to stable RRMS [76]. SPMS patients displayed a decreased frequency of M-MDSCs and G-MDSCs compared to HCs (*p* < 0.05) [76].

Additionally, the analysis of the T-cell regulatory function of M-MDSCs demonstrated T-cell suppressive capacity in RRMS and HCs, while M-MDSCs of SPMS promoted autologous T-cell proliferation, which aligned with a differential cytokine profile compared to RRMS and HCs [76].

This study documented a phenotypic and functional transition of MDSCs across distinct clinical stages of MS, implying their potential as therapeutic targets for mitigating the progression of MS [76]. The observed divergence within the subset of MS disease could potentially be attributed to dissimilarities in the two cohorts of subjects that were scrutinized (*e.g*., genetic variations, disparities in disease duration, and prior immunomodulatory interventions) [76].

In a study by Cantoni *et al*. [77], the authors showed a deficient M-MDSCs function in patients with MS and investigated human M-MDSCs function in suppressing T-cell activity [77]. Here, the evidence was provided that micro-RNA-223 expression in MDSCs from patients with MS is responsible for the reduction in M-MDSCs suppression. Targeting micro-RNA-223 could boost M-MDSCs suppression of T-cell proliferation *via* STAT3 activation [77]. Additionally, they also found increased M-MDSCs suppressive functions after glucocorticoid treatment, but this effect was STAT3 independent [77].

In the aforementioned study by Ortega *et al*. [58], M-MDSCs presence within white matter lesions of MS patients was also investigated. High inflammatory areas, such as active lesions and lesions with paramagnetic rim, showed an increased level of M-MDSCs infiltration compared to inactive lesions and active lesions with hypocellular lesion centers, laying the basis for their study as a future biomarker for disease severity.

## CONCLUSION

**Fig. (2):**
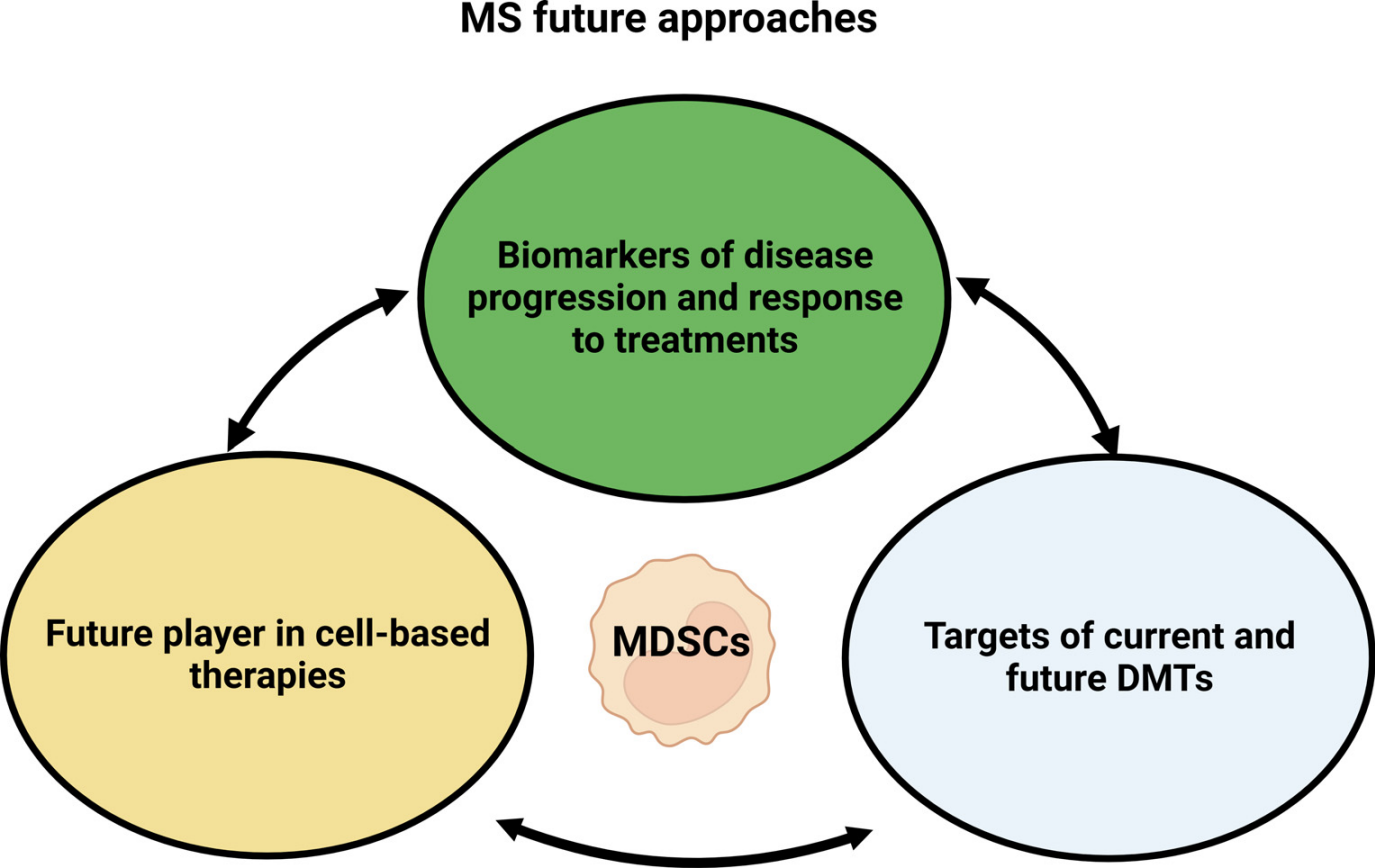
MDSCs and MS future approaches.

The immunosuppressive functions of MDSCs can be dysregulated in MS, leading to an exacerbation of the autoimmune response and disease progression. Therefore, targeting MDSCs could be a promising therapeutic approach for MS (Fig. **2**).

In future MS approaches, MDSCs could be employed as biomarkers of disease progression and response to treatment.

It would be of interest to define their role in the initial phases of the disease or in chronic phases so that their levels and characterization in different disease stages could inform clinical practice.

Then, they could be employed for cell-based therapies. As discussed in the previous sections, antigen-specific peptide immunotherapy, which aims to restore tolerance while avoiding the use of non-specific immunosuppressive drugs, is a promising approach for autoimmune diseases, but the cellular mechanisms behind successful therapy remain poorly understood.

MDSCs have been studied intensively in the field of cancer and, to a lesser extent, in autoimmunity. Because of their suppressive effect on the immune system in cancer, the development of MDSCs and their interaction with CD4+ T cells could be beneficial for antigen-specific immunotherapy.

The precise categorization of the G-MDSCs elucidated in this study as either a distinct subset of neutrophils or undifferentiated MDSCs remains unclear.

Regardless of the differentiation status attributed to G-MDSCs within the autoimmune context, these findings substantiate a regulatory function for G-MDSCs exhibiting characteristics akin to “neutrophils” in ameliorating autoimmune-driven inflammation. Further phenotypic characterization of the MDSCs is needed to reassess their potential in the regulation of immune responses. However, it remained unclear whether MDSCs display any therapeutic potential in MS and how this therapy could modulate different aspects of the disease, as seen for gut microbiota composition.

Collectively, all the described studies revealed a pivotal role for MDSCs in the regulation of MS.

Various strategies for modulating MDSCs have been investigated, including the use of pharmacological agents, biological agents, and adoptive transfer of exogenous MDSCs. As mediated by studies in other fields, MDSCs could be proposed as a therapeutic strategy for MS either through the modulation of endogenous MDSCs or through the adoptive transfer of exogenous MDSCs. One approach to modulating endogenous MDSCs is using pharmacological agents that can induce the expansion of MDSCs or enhance their immunosuppressive functions. For example, all-trans retinoic acid has been shown to induce the expansion of MDSCs and promote their immunosuppressive in a mouse model of MS [65]. Another approach to modulating MDSCs in MS is through the adoptive transfer of exogenous MDSCs. This approach involves the isolation and expansion of MDSCs from a donor, followed by the infusion of these cells into a recipient. The goal of this approach is to enhance the immunosuppressive functions of MDSCs and promote their recruitment to the CNS to suppress auto-reactive T-cell responses.

However, there are several challenges associated with the adoptive transfer of MDSCs. One challenge is the need for an adequate number of MDSCs for infusion, which requires extensive *ex vivo* expansion. Another challenge is the potential for the infused MDSCs to lose their immunosuppressive functions in the inflammatory environment of the CNS. Additionally, there is a risk of adverse events associated with the infusion of exogenous cells, such as an increased risk of infection or the potential for graft-*versus*-host disease.

These approaches have shown promise in preclinical models of MS, and some have progressed to clinical trials. However, there are still several challenges associated with these approaches, including the need for a better understanding of the complex mechanisms regulating MDSCs function and the potential for adverse events associated with the infusion of exogenous cells. Nevertheless, targeting MDSCs represents an exciting avenue for the development of novel therapeutics for MS.

Furthermore, MDSCs may serve as a valuable parameter for discerning patients with an increased likelihood of exhibiting suboptimal responses to DMTs. This observation opens avenues for the identification of robust biomarkers that enable the monitoring or prediction of efficacious responses to DMTs, thereby facilitating their precise and well-informed prescription for patient management in the foreseeable future.

The plasticity of MDSCs underscores their significance in regulating immune responses and their potential as therapeutic targets in autoimmune diseases and other inflammatory conditions. Further research is needed to unravel the precise mechanisms that dictate the MDSC's contribution to immune regulation and tissue homeostasis. It's important to note that the differentiation of MDSCs into macrophages or DCs is not fully understood, and the factors that govern this process in various disease contexts are still being explored.

However, despite the promise of MDSCs as therapeutic agents, several challenges need to be addressed. Overcoming the blood-brain barrier to effectively deliver MDSCs to the CNS remains a significant hurdle. Additionally, the intricate interactions of MDSCs with other immune cells and their specific roles in different stages of MS progression require further investigation for successful implementation.

Nonetheless, with continued research and technological advancements, harnessing the regulatory capabilities of MDSCs could lead to ground-breaking MS therapies.

Targeted interventions involving MDSCs may offer new avenues for disease management, potentially improving the quality of life for individuals living with MS and providing hope for a future where the course of the disease can be effectively controlled.

## Figures and Tables

**Appendix (1B):** Journal_wise_ms_data_reviewer_report.

**PMID**	**Title**	**Authors**	**Citation**	**First Author**	**Journal/ Book**	**Publication Year**	**Create Date**	**PMCID**	**NIHMS ID**	**DOI**
36403026	Peripheral myeloid-derived suppressor cells are good biomarkers of the efficacy of fingolimod in multiple sclerosis	Camacho-Toledano C, Machín-Díaz I, Calahorra L, Cabañas-Cotillas M, Otaegui D, Castillo-Triviño T, Villar LM, Costa-Frossard L, Comabella M, Midaglia L, García-Domínguez JM, García-Arocha J, Ortega MC, Clemente D.	J Neuroinflammation. 2022 Nov 19 19(1): 277. doi: 10.1186/s12974-022-02635-3.	Camacho-Toledano C	J Neuroinflammation	2022	19/11/22	PMC9675277	-	10.1186/s12974-022-02635-3
36184742	Myeloid-derived suppressor cells prevent disruption of the gut barrier, preserve microbiota composition, and potentiate immunoregulatory pathways in a rat model of experimental autoimmune encephalomyelitis	Radojević D, Bekić M, Gruden-Movsesijan A, Ilić N, Dinić M, Bisenić A, Golić N, Vučević D, Đokić J, Tomić S.	Gut Microbes. 2022 Jan-Dec 14(1): 2127455. doi: 10.1080/19490976.2022.2127455.	Radojević D	Gut Microbes	2022	02/10/22	PMC9543149	-	10.1080/19490976.2022.2127455
36159850	Maturation of circulating Ly6C(hi)CCR2(+) monocytes by mannan-MOG induces antigen-specific tolerance and reverses autoimmune encephalomyelitis	Dagkonaki A, Papalambrou A, Avloniti M, Gkika A, Evangelidou M, Androutsou ME, Tselios T, Probert L.	Front Immunol. 2022 Sep 913: 972003. doi: 10.3389/fimmu.2022.972003. eCollection 2022.	Dagkonaki A	Front Immunol	2022	26/09/22	PMC9501702	-	10.3389/fimmu.2022.972003
35678041	Treatment of EAE mice with Treg, G-MDSC and IL-2: a new insight into cell therapy for multiple sclerosis	Ghorbani MM, Farazmandfar T, Abediankenari S, Hassannia H, Maleki Z, Shahbazi M.	Immunotherapy. 2022 Jul14(10): 789-798. doi: 10.2217/imt-2021-0045. Epub 2022 Jun 8.	Ghorbani MM	Immunotherapy	2022	09/06/22	-	-	10.2217/imt-2021-0045
35273601	Immunological Subsets Characterization in Newly Diagnosed Relapsing-Remitting Multiple Sclerosis	D'Amico E, Zanghì A, Parrinello NL, Romano A, Palumbo GA, Chisari CG, Toscano S, Raimondo FD, Zappia M, Patti F.	Front Immunol. 2022 Feb 22 13: 819136. doi: 10.3389/fimmu.2022.819136. eCollection 2022.	D'Amico E	Front Immunol	2022	11/03/22	PMC8902351	-	10.3389/fimmu.2022.819136
33217041	Myeloid-derived suppressor cells support remyelination in a murine model of multiple sclerosis by promoting oligodendrocyte precursor cell survival, proliferation, and differentiation	Melero-Jerez C, Fernández-Gómez B, Lebrón-Galán R, Ortega MC, Sánchez-de Lara I, Ojalvo AC, Clemente D, de Castro F.	Glia. 2021 Apr69(4): 905-924. doi: 10.1002/glia.23936. Epub 2020 Nov 20.	Melero-Jerez C	Glia	2021	20/11/20	PMC7894183	-	10.1002/glia.23936
32709481	Type I IFN signaling in T regulatory cells modulates chemokine production and myeloid derived suppressor cells trafficking during EAE	Tanwar S, Oguz C, Metidji A, Dahlstrom E, Barbian K, Kanakabandi K, Sykora L, Shevach EM.	J Autoimmun. 2020 Dec115: 102525. doi: 10.1016/j.jaut.2020.102525. Epub 2020 Jul 22.	Tanwar S	J Autoimmun	2020	26/07/20	PMC7712497	NIHMS1648446	10.1016/j.jaut.2020.102525
32278882	The proportion of myeloid-derived suppressor cells in the spleen is related to the severity of the clinical course and tissue damage extent in a murine model of multiple sclerosis	Melero-Jerez C, Alonso-Gómez A, Moñivas E, Lebrón-Galán R, Machín-Díaz I, de Castro F, Clemente D.	Neurobiol Dis. 2020 Jul; 140: 104869. doi: 10.1016/j.nbd.2020.104869. Epub 2020 Apr 9.	Melero-Jerez C	Neurobiol Dis	2020	13/04/20	-	-	10.1016/j.nbd.2020.104869
30958568	Remote control of T(h) 17 responses: The lung-CNS axis during EAE	Vijitha N, Engel DR.	J Leukoc Biol. 2019 May; 105(5): 827-828. doi: 10.1002/JLB.1CE0219-072R. Epub 2019 Apr 8.	Vijitha N	J Leukoc Biol	2019	09/04/19	-	-	10.1002/JLB.1CE0219-072R
30798007	The presence and suppressive activity of myeloid-derived suppressor cells are potentiated after interferon-β treatment in a murine model of multiple sclerosis	Melero-Jerez C, Suardíaz M, Lebrón-Galán R, Marín-Bañasco C, Oliver-Martos B, Machín-Díaz I, Fernández Ó, de Castro F, Clemente D.	Neurobiol Dis. 2019 Jul; 127: 13-31. doi: 10.1016/j.nbd.2019.02.014. Epub 2019 Feb 21.	Melero-Jerez C	Neurobiol Dis	2019	25/02/19	-	-	10.1016/j.nbd.2019.02.014
30762897	Frontline Science: Induction of experimental autoimmune encephalomyelitis mobilizes Th17-promoting myeloid derived suppressor cells to the lung	Glenn JD, Liu C, Whartenby KA.	J Leukoc Biol. 2019 May; 105(5): 829-841. doi: 10.1002/JLB.4HI0818-335R. Epub 2019 Feb 14.	Glenn JD	J Leukoc Biol	2019	15/02/19	-	-	10.1002/JLB.4HI0818-335R
30374128	Myeloid-derived suppressor cells control B cell accumulation in the central nervous system during autoimmunity	Knier B, Hiltensperger M, Sie C, Aly L, Lepennetier G, Engleitner T, Garg G, Muschaweckh A, Mitsdörffer M, Koedel U, Höchst B, Knolle P, Gunzer M, Hemmer B, Rad R, Merkler D, Korn T.	Nat Immunol. 2018 Dec; 19(12): 1341-1351. doi: 10.1038/s41590-018-0237-5. Epub 2018 Oct 29.	Knier B	Nat Immunol	2018	31/10/18	PMC6241855	EMS79534	10.1038/s41590-018-0237-5
30123217	Cannabidiol Attenuates Experimental Autoimmune Encephalomyelitis Model of Multiple Sclerosis Through Induction of Myeloid-Derived Suppressor Cells	Elliott DM, Singh N, Nagarkatti M, Nagarkatti PS.	Front Immunol. 2018 Aug 3; 9: 1782. doi: 10.3389/fimmu.2018.01782. eCollection 2018.	Elliott DM	Front Immunol	2018	21/08/18	PMC6085417	-	10.3389/fimmu.2018.01782
30068593	Glatiramer Acetate Enhances Myeloid-Derived Suppressor Cell Function *via* Recognition of Paired Ig-like Receptor B	van der Touw W, Kang K, Luan Y, Ma G, Mai S, Qin L, Bian G, Zhang R, Mungamuri SK, Hu HM, Zhang CC, Aaronson SA, Feldmann M, Yang WC, Chen SH, Pan PY.	J Immunol. 2018 Sep 15; 201(6): 1727-1734. doi: 10.4049/jimmunol.1701450. Epub 2018 Aug 1.	van der Touw W	J Immunol	2018	03/08/18	PMC6379207	NIHMS981673	10.4049/jimmunol.1701450
29569304	Phenotypic and functional alterations of myeloid-derived suppressor cells during the disease course of multiple sclerosis	Iacobaeus E, Douagi I, Jitschin R, Marcusson-Ståhl M, Andrén AT, Gavin C, Lefsihane K, Davies LC, Mougiakakos D, Kadri N, Le Blanc K.	Immunol Cell Biol. 2018 Sep; 96(8): 820-830. doi: 10.1111/imcb.12042. Epub 2018 Apr 19.	Iacobaeus E	Immunol Cell Biol	2018	24/03/18	-	-	10.1111/imcb.12042
28140447	Myeloid-derived suppressor cells mediate tolerance induction in autoimmune disease	Wegner A, Verhagen J, Wraith DC.	Immunology. 2017 May; 151(1): 26-42. doi: 10.1111/imm.12718. Epub 2017 Mar 2.	Wegner A	Immunology	2017	01/02/17	PMC5382345	-	10.1111/imm.12718
27693617	Myeloid-derived suppressor cells expressing a self-antigen ameliorate experimental autoimmune encephalomyelitis	Casacuberta-Serra S, Costa C, Eixarch H, Mansilla MJ, López-Estévez S, Martorell L, Parés M, Montalban X, Espejo C, Barquinero J.	Exp Neurol. 2016 Dec; 286: 50-60. doi: 10.1016/j.expneurol.2016.09.012. Epub 2016 Sep 28.	Casacuberta-Serra S	Exp Neurol	2016	04/10/16	-	-	10.1016/j.expneurol.2016.09.012
27704281	Mir-223 regulates the number and function of myeloid-derived suppressor cells in multiple sclerosis and experimental autoimmune encephalomyelitis	Cantoni C, Cignarella F, Ghezzi L, Mikesell B, Bollman B, Berrien-Elliott MM, Ireland AR, Fehniger TA, Wu GF, Piccio L.	Acta Neuropathol. 2017 Jan; 133(1): 61-77. doi: 10.1007/s00401-016-1621-6. Epub 2016 Oct 4.	Cantoni C	Acta Neuropathol	2017	06/10/16	PMC5423756	NIHMS857015	10.1007/s00401-016-1621-6
24709559	The synthetic retinoid Am80 delays recovery in a model of multiple sclerosis by modulating myeloid-derived suppressor cell fate and viability	Moliné-Velázquez V, Ortega MC, Vila del Sol V, Melero-Jerez C, de Castro F, Clemente D.	Neurobiol Dis. 2014 Jul; 67: 149-64. doi: 10.1016/j.nbd.2014.03.017. Epub 2014 Apr 5.	Moliné-Velázquez V	Neurobiol Dis	2014	09/04/14	-	-	10.1016/j.nbd.2014.03.017
23997223	Inhibition of endogenous activated protein C attenuates experimental autoimmune encephalomyelitis by inducing myeloid-derived suppressor cells	Alabanza LM, Esmon NL, Esmon CT, Bynoe MS.	J Immunol. 2013 Oct 1; 191(7): 3764-77. doi: 10.4049/jimmunol.1202556. Epub 2013 Aug 30.	Alabanza LM	J Immunol	2013	03/09/13	PMC3800123	NIHMS512853	10.4049/jimmunol.1202556
23637087	Developmental maturation of innate immune cell function correlates with susceptibility to central nervous system autoimmunity	Hertzenberg D, Lehmann-Horn K, Kinzel S, Husterer V, Cravens PD, Kieseier BC, Hemmer B, Brück W, Zamvil SS, Stüve O, Weber MS.	Eur J Immunol. 2013 Aug; 43(8): 2078-88. doi: 10.1002/eji.201343338. Epub 2013 Jun 3.	Hertzenberg D	Eur J Immunol	2013	03/05/13	PMC4136766	NIHMS513549	10.1002/eji.201343338
23345328	Activated invariant NKT cells control central nervous system autoimmunity in a mechanism that involves myeloid-derived suppressor cells	Parekh VV, Wu L, Olivares-Villagómez D, Wilson KT, Van Kaer L.	J Immunol. 2013 Mar 1; 190(5): 1948-60. doi: 10.4049/jimmunol.1201718. Epub 2013 Jan 23.	Parekh VV	J Immunol	2013	25/01/13	PMC3577977	NIHMS432530	10.4049/jimmunol.1201718
23034169	Mouse CD11b+Gr-1+ myeloid cells can promote Th17 cell differentiation and experimental autoimmune encephalomyelitis	Yi H, Guo C, Yu X, Zuo D, Wang XY.	J Immunol. 2012 Nov 1; 189(9): 4295-304. doi: 10.4049/jimmunol.1200086. Epub 2012 Oct 3.	Yi H	J Immunol	2012	05/10/12	PMC3478426	NIHMS404830	10.4049/jimmunol.1200086
22210912	Crucial role of granulocytic myeloid-derived suppressor cells in the regulation of central nervous system autoimmune disease	Ioannou M, Alissafi T, Lazaridis I, Deraos G, Matsoukas J, Gravanis A, Mastorodemos V, Plaitakis A, Sharpe A, Boumpas D, Verginis P.	J Immunol. 2012 Feb 1; 188(3): 1136-46. doi: 10.4049/jimmunol.1101816. Epub 2011 Dec 30.	Ioannou M	J Immunol	2012	03/01/12	-	-	10.4049/jimmunol.1101816
21507122	Myeloid-derived suppressor cells limit the inflammation by promoting T lymphocyte apoptosis in the spinal cord of a murine model of multiple sclerosis	Moliné-Velázquez V, Cuervo H, Vila-Del Sol V, Ortega MC, Clemente D, de Castro F.	Brain Pathol. 2011 Nov; 21(6): 678-91. doi: 10.1111/j.1750-3639.2011.00495.x. Epub 2011 Jun 9.	Moliné-Velázquez V	Brain Pathol	2011	22/04/11	PMC8094047	-	10.1111/j.1750-3639.2011.00495.x
19890055	Innate immune CD11b+Gr-1+ cells, suppressor cells, affect the immune response during Theiler's virus-induced demyelinating disease	Bowen JL, Olson JK.	J Immunol. 2009 Dec 1; 183(11): 6971-80. doi: 10.4049/jimmunol.0902193. Epub 2009 Nov 4.	Bowen JL	J Immunol	2009	06/11/09	-	-	10.4049/jimmunol.0902193
19196868	Circulating Ly-6C+ myeloid precursors migrate to the CNS and play a pathogenic role during autoimmune demyelinating disease	King IL, Dickendesher TL, Segal BM.	Blood. 2009 Apr 2; 113(14): 3190-7. doi: 10.1182/blood-2008-07-168575. Epub 2009 Feb 5.	King IL	Blood	2009	07/02/09	PMC2665891	-	10.1182/blood-2008-07-168575
29484707	2-AG limits Theiler's virus induced acute neuroinflammation by modulating microglia and promoting MDSCs	Mecha M, Feliú A, Machín I, Cordero C, Carrillo-Salinas F, Mestre L, Hernández-Torres G, Ortega-Gutiérrez S, López-Rodríguez ML, de Castro F, Clemente D, Guaza C.	Glia. 2018 Jul; 66(7): 1447-1463. doi: 10.1002/glia.23317. Epub 2018 Feb 27.	Mecha M	Glia	2018	28/02/18	-	-	10.1002/glia.23317
37243699	Central and peripheral myeloid-derived suppressor cell-like cells are closely related to the clinical severity of multiple sclerosis	Ortega MC, Lebrón-Galán R, Machín-Díaz I, Naughton M, Pérez-Molina I, García-Arocha J, Garcia-Dominguez JM, Goicoechea-Briceño H, Vila-Del Sol V, Quintanero-Casero V, García-Montero R, Galán V, Calahorra L, Camacho-Toledano C, Martínez-Ginés ML, Fitzgerald DC, Clemente D.	Acta Neuropathol. 2023 Aug; 146(2): 263-282. doi: 10.1007/s00401-023-02593-x. Epub 2023 May 27.	Ortega MC	Acta Neuropathol	2023	27/05/23	PMC10329064	-	10.1007/s00401-023-02593-x
32301489	NAD+ attenuates experimental autoimmune encephalomyelitis through induction of CD11b+ gr-1+ myeloid-derived suppressor cells	Wang JL, Li B, Tan GJ, Gai XL, Xing JN, Wang JQ, Quan MY, Zhang N, Guo L.	Biosci Rep. 2020 Apr 30; 40(4): BSR20200353. doi: 10.1042/BSR20200353.	Wang JL	Biosci Rep	2020	18/04/20	PMC7182659	-	10.1042/BSR20200353
36082828	Pseudolycorine chloride ameliorates Th17 cell-mediated central nervous system autoimmunity by restraining myeloid-derived suppressor cell expansion	Zhang G, Zhu X, Yang F, Li J, Leng X, Mo C, Li L, Wang Y.	Pharm Biol. 2022 Dec; 60(1): 899-908. doi: 10.1080/13880209.2022.2063344.	Zhang G	Pharm Biol	2022	09/09/22	PMC9467541	-	10.1080/13880209.2022.2063344
17911608	CD11b+Ly-6C(hi) suppressive monocytes in experimental autoimmune encephalomyelitis	Zhu B, Bando Y, Xiao S, Yang K, Anderson AC, Kuchroo VK, Khoury SJ.	J Immunol. 2007 Oct 15; 179(8): 5228-37. doi: 10.4049/jimmunol.179.8.5228.	Zhu B	J Immunol	2007	04/10/07	-	-	10.4049/jimmunol.179.8.5228
21824867	Plasticity of Ly-6C(hi) myeloid cells in T cell regulation	Zhu B, Kennedy JK, Wang Y, Sandoval-Garcia C, Cao L, Xiao S, Wu C, Elyaman W, Khoury SJ.	J Immunol. 2011 Sep 1; 187(5): 2418-32. doi: 10.4049/jimmunol.1100403. Epub 2011 Aug 8.	Zhu B	J Immunol	2011	10/08/11	PMC3159773	NIHMS310532	10.4049/jimmunol.1100403
33046864	Prolonged residence of an albumin-IL-4 fusion protein in secondary lymphoid organs ameliorates experimental autoimmune encephalomyelitis	Ishihara A, Ishihara J, Watkins EA, Tremain AC, Nguyen M, Solanki A, Katsumata K, Mansurov A, Budina E, Alpar AT, Hosseinchi P, Maillat L, Reda JW, Kageyama T, Swartz MA, Yuba E, Hubbell JA.	Nat Biomed Eng. 2021 May; 5(5): 387-398. doi: 10.1038/s41551-020-00627-3. Epub 2020 Oct 12.	Ishihara A	Nat Biomed Eng	2021	13/10/20	-	-	10.1038/s41551-020-00627-3

**Table 1:** MDSCs in MS disease models.

**Authors, Year, Journal**	**Investigated Cell Type(s)**	**Pre-clinical Model Details**	**Summarized Findings**	**References**
Ghorbani *et al*. 2022, Immunotherapy	**G-MDSCs**	G-MDSCs and Tregs were isolated from the spleen of two healthy mice. The frequency of cells after purification was confirmed using flow cytometry. Before injection, the Tregs were incubated with IL-2 to increase FOXP3 expression. 40 mice were randomly divided into eight groups of five mice as follows: 1) normal control group (recipient of PBS). 2) EAE group (MOG induction). 3) posttreatment groups (treatment injected 14 DPI) including: A. EAE + Treg. B. EAE + G-MDSC. C. EAE + IL-2. 4) pretreatment groups (treatment injected 1 DBI) including: A. Treg + EAE. B. G-MDSC + EAE. C. IL-2 + EAE. Brain and spleen samples collected after sacrifice.*Ex vivo* tissue and cells analysis performed with immunohistochemistry, immunostaining and flow cytometry. Serum concentration of IL-17 20 DPI measured with ELISA kit.	• Groups 3A, 3B and 3C showed ↓ inflammation and demyelination compared to untreated mice. • Groups 4A, 4B and 4C showed no inflammation and demyelination in the brain. • Pre and post treatment of EAE mice with Treg, G-MDSCs and IL-2 ↓ Th17 cells in the spleen of EAE mice and ↓ IL-17 serum concentration compared to untreated EAE mice. • Pre and post treatment of EAE mice with G-MDSCs ↑ G-MDSCs in the spleen of treated mice.	[43]
Melero-Jerez *et al*. 2021, Glia	**M-MDSCs**	**EAE Mice Model** M-MDSCs and OPCs isolated from EAE animals tissue samples, analyzed with immunohistochemistry, and used for *in vitro* cultivation. Assessment of M-MDSCs effects on OPCs performed through survival assay, proliferation assay, chemotaxis chambers and differentiation assay. Soluble factors produced by M-MDSCs determined through MDSC-conditioned medium proteome profiling.	• M-MDSCs levels in the spinal cord of EAE mice are directly correlated with OPCs distribution in demyelinated lesions. • M-MDSCs promote OPCs differentiation towards myelinating phenotypes, mainly through osteopontin.	[44]
Melero-Jerez *et al*. 2020, Neurobiol Dis.	**M-MDSCs**	**EAE Mice Model** Clinical signs obtained from the observational evaluation of the functional behavior of each mouse during their clinical course before its sacrifice. Spinal cord and spleen of each mouse collected after sacrifice. *Ex vivo* cell analysis performed with immunohistochemistry and flow cytometry.	M-MDSCs splenic levels are directly correlated with: • preservation of myelin and axons. • T cell apoptosis within the CNS. • milder disease course.	[45]
Elliot *et al*. 2018, Front Immunol.	**G-MDSCs**	**EAE Mice Model** CBD treatment, vehicle treatment and CBD + RB6-8C5 of EAE mice was performed, resulting in EAE-CBD mice group, EAE-VEH mice control group and CBD-EAE mice with MDSC ablation group. MDSCs isolated from the peritoneal cavity of EAE-CBD mice analyzed with flow cytometry. CNS infiltrating cells, isolated from whole brain and spinal cord homogenates of each group, analyzed with flow cytometry.	• CBD treatment leads to ↑ G-MDSCs in EAE mice and attenuates EAE disease progression by ↓ MOG-specific T cell proliferation in the CNS. • Attenuation of EAE by CBD treatment can be reversed with G-MDSCs depletion.	[46]
Mecha *et al*. 2018, Glia	**M-MDSCs**	**TMEV-IDD Model** TMEV mice were treated daily for seven consecutive days (0-7 DPI) with 2-AG + UCM-03025 or vehicle alone. CNS and spleen samples collected after sacrifice. *Ex vivo* tissue and cells analysis performed with immunohistochemistry, immunostaining and flow cytometry. M-MDSCs isolated from the spleen of vehicle or 2-AG-injected mice co-cultured with activated T cells.	• 2-AG treatment ↑ M-MDSCs into the CNS. • 2-AG treatment ↓ CD4+ T cells and ↑ Apoptotic CD4+ T into the CNS. • 2-AG treatment ↓ microglial activation. • 2-AG treatment ↓ M-MDSCs in the LN and ↑ M-MDSCs in the spleen. • M-MDSCs isolated from the spleen of vehicle or 2-AG-injected mice ↓↓ activated T cell proliferation. • Early 2-AG treatment ↓ motor deficits of mice at the chronic phase of TMEV disease.	[47]
-	-	TMEV mice were treated for seven consecutive days (0-7 DPI) with the CB1 receptor antagonist AM251 or the CB2 receptor antagonist AM630 30 min before the administration of 2-AG, UCM-03025 or vehicle alone. CNS and spleen samples collected after sacrifice. *Ex vivo* tissue and cells analysis performed with immunohistochemistry, immunostaining, and flow cytometry. Total RNA from the prefrontal cortex was extracted and RT-PCR was performed on the cDNA to analyze anti-inflammatory mediators, pro-inflammatory mediators, chemokines.	• 2-AG administration + CB2 antagonism ↑ microglial activation. • 2-AG administration + CB1 and CB2 antagonism reversed downregulation of chemokine expression resulting from 2-AG administration. • 2-AG administration + CB1 and CB2 antagonism ↑ total cells number in both the LN and spleen.	-
Bowen *et al*. 2009, J. Immunol.	**M-MDSC, G-MDSCs**	**TMEV-IDD Model** TMEV mice model were treated with anti-Gr-1 or IgG (control group) on -1, 0, 1, and 2 DPI. Spinal cord and spleen of each mouse collected after sacrifice. *Ex vivo* cell analysis performed with immunohistochemistry, flow cytometry and T cells assays. RNA extracted from isolated cells and analyzed with RT-PCR.	• M-MDSCs (and not G-MDSCs) ↑ into the CNS during the innate immune response to TMEV in the control TMEV mice group. • Anti-Gr-1 treated mice showed: • ↓ Development and progression of demyelinating disease. • ↑ Expression of proinflammatory cytokines in the CNS. • ↓ Expression of IL-10 in the CNS. • ↑ CD4+ T cell and CD8+ T cell responses against virus, enabling a more effective virus clearance (confirmed by ↓ virus load in the CNS).	[48]
Wegner *et al*. 2017, Immunology	**G-MDSCs**	**EAE in TCR-transgenic mice model** Mice underwent EDI with MBP_Ac1-9_ 4K and MBP_Ac1-9_ 4Y. Changes in the quantity, phenotype and function of G-MDSCs during EDI evaluated through flow cytometry and *in vitro* techniques. Myelin basic protein‐specific CD4+ T cells were polarized *in vitro* under normal Th1 conditions either with or without the addition of G‐MDSC, before adoptive transfer to TCR-transgenic mice recipients to induce a passive form of EAE.	• G-MDSCs levels ↑ during EDI • G-MDSCs developed during EDI suppress CD4+ T cells *in vitro* through cell-contact-dependent manner mediated by Arg-1. • The addition of G‐MDSC during Th1 polarization *in vitro* ↓ EAE severity and ↑ recovery from disease in TCR-transgenic mice recipients of Th1 cells.	[49]
Casacuberta-Serra *et al*., 2016, Exp Neurol.	**M-MDSCs, G-MDSCs**	**EAE Mice Model** Antigen-expressing and control MDSCs (19.7 ± 3.2% M-MDSCs; 68.7 ± 4.8% G-MDSCs) were incubated with splenocytes from EAE mice for 18 h. Apoptosis measured in the different T cell subpopulations by flow cytometry.	• Antigen-expressing M and G-MDSCs ↑ apoptosis in CD4+ cells.	[50]
Ioannou *et al*. 2012, J Immunol	**G-MDSCs**	**EAE Mice Model** G-MDSCs sorted from spleens of EAE mice and: 1) Used for phenotypic characterization. 2) Treated with IFN-γ (to ↑ PD-L1 expression) and co-cultured with CD4+CD25− T cells (sorted from spleens of naive WT or PD-1−/− mice), to assess the role of PD-L1/PD-1 interactions *in vitro*. 3) Adoptively transferred into different EAE mice on 4 and 7 DPI. CNS and spleen samples collected after sacrifice. *Ex vivo* tissue and cells analysis performed with immunofluorescence and flow cytometry. EAE mice or CFA immunized (untreated) mice adoptively transferred with PD-L1−/− G-MDSCs, to assess the role of PD-L1/PD-1 interactions *in vivo*.	• G-MDSCs from EAE mice showed ↑ expression of PD-L1. • PD-L1/PD-1 interactions between G-MDSCs and T cells reportedly deliver coinhibitory signals, leading to ↓ of T cell responses both *in vitro* and *in vivo*. • G-MDSCs transfer into EAE mice ↓ inflammatory lesions and demyelination, ameliorating EAE.	[51]
Dagkonaki *et al*. 2022, Front. Immunol	**M-MDSCs**	**EAE Mice Model** Mice received i.d. OM-MOG injections starting at clinical score 2 (mild hind limb weakness), and thereafter every 2 days. Circulating monocytes subsets determined through flow cytometry. Spinal cord collected after sacrifice. *Ex vivo* cell analysis performed with immunofluorescence staining and flow cytometry.	• OM-MOG injections ↑ MHCII and ↑ PD-L1 production by M-MDSCs. • OM-MOG injections prevent M-MDSCs migration to the spinal cord.	[52]
		Anti‐CD3/CD28 stimulated splenic CD3+ cells from control mice co-cultured with M-MDSCs isolated from the spinal cord of EAE mice with a peak clinical score.	• M-MDSCs ↑ CD3+ apoptosis.	
Ishihara *et al*. 2021, Nat. Biomed. Eng.	**M-MDSCs, G-MDSCs**	**EAE Mice Model** EAE mice injected every other day for 10 days with PBS i.p., WT IL-4 i.p. or SA–IL-4 (i.p. and s.c.) starting 8 DPI. Alternatively, FTY7208 administered orally every day from day 8 after immunization. Cells from the dLNs and spinal cords were isolated 17 days after immunization and analyzed by flow cytometry. Histology of spinal cord sections was also performed: myelin expression was detected by immunohistochemistry with anti-myelin basic protein antibody.	• I.p. injection of SA–IL-4 and FTY720 both ↓ EAE development and ↓ immune-cell infiltration into the spinal cord. • SA–IL-4 s.c. ↓↓ detectable demyelination of the spinal cord. • SA–IL-4 ↑ G-MDSCs, ↓ M-MDSCs and ↓ Th17 CD4+ T. • SA–IL-4 ↑ expression of PD-1 on both CD4+ and CD8+ T cells.	[53]
		**EAE Mice Model** EAE mice were injected with PBS, WT IL-4 or SA–IL-4 s.c. on 8, 10 and 12 DPI (PBS treatment group, n= 7; all other groups, n= 6). The spinal cords and spleens of the mice were isolated on day 13 and the immune cells were analyzed by flow cytometry.	SA–IL-4 ↑ expression levels of PD-L1 and ↑ frequency of PD-L1-expressing cells on both M-MDSCs and G-MDSCs.	
Moliné‐Velázquez *et al*. 2011, Brain Pathol.	**M-MDSCs**	**EAE Mice Model** Spinal cord dissected out of EAE mice at each of following the stages: 15, 25, 35 and 63 DPI. MDSCs subsets and T cells subsets analyzed in 12 sections from all the demyelinated areas of the spinal cord of the sacrificed mice, by immunohistochemical analysis.	M-MDSCs are Arg-I+ and: • ↑ during the active phase of EAE (15 DPI); • ↓ when the immune response becomes limited (25 DPI); • Disappear during the chronic phase (35 DPI and onwards).	[54]
Wang *et al*. 2020, Biosci Rep.	**G-MDSCs**	**EAE Mice Model** EAE mice model were treated with NAD+ or PBS daily from the day of inoculation. Spinal cord and spleen of each mouse collected after sacrifice. *Ex vivo* cell analysis performed with immunofluorescent staining, flow cytometry, ELISA, and western blot.	• NAD+ treatment ↑ G-MDSCs in the spleen. • NAD+ treatment ↑ Arg-1 in spleen, spinal cord, and serum. • NAD+ treatment ↓ IFN-γ and IL-17 levels and ↑ IL-13 in serum, regulating inflammatory response of Th1 and Th2 cells. • NAD+ treatment diminished clinical scores of EAE and delayed disease onset.	[55]
Zhu *et al*. 2007, J. Immunol.	**M-MDSCs**	**EAE Mice Model** Splenocytes were isolated from EAE mice and spinal cord tissues were dissected out after sacrifice. *Ex vivo* cell analysis performed with flow cytometry and staining kits. RNA was extracted from isolated cells and analyzed with RT-PCR.	• M-MDSCs ↑ in the spleen after EAE induction and suppress CD4 T cell proliferation *in vitro*.	[56]
Zhu *et al*. 2011, J. Immunol	**M-MDSCs**	**EAE Mice Model** Spinal cord and spleen of each mouse collected after sacrifice. *Ex vivo* cell analysis performed with immunofluorescence staining and flow cytometry.	• M-MDSCs in the CNS are ↑ from disease onset to peak and switch their function from antigen presentation to T cell suppression. • Transfer of activated M-MDSCs enhances T cell apoptosis in the CNS and suppresses EAE.	[57]
Ortega *et al*. 2023, Acta Neuropat.	**M-MDSCs**	**EAE Mice Model** Blood and spinal cord of each mouse collected after sacrifice at peak disease. *Ex vivo* cell and tissue analysis performed with immunohistochemistry and flow cytometry. M-MDSCs isolated from peripheral blood co-cultured with T-cells isolated from the spleen of different EAE mice.	• ↑ M-MDSCs at disease onset correlate with the future milder disease severity. • Peripheral M-MDSCs ↓ CD4 and CD8 T cell proliferation *in vitro*.	[58]
Glenn *et al*. 2019, J Leukoc Biol.	**M-MDSCs, G-MDSCs**	**EAE Mice Model** Mice sacrificed at indicated time points after immunization and whole lungs harvested. *Ex vivo* cell analysis performed with flow cytometry.	• G-MDSCs levels in the lungs ↑ during EAE, with a peak at 7 DPI and 10 DPI. • M-MDSCs were ↓↓ in the lungs compared to G-MDSCs.	[59]
		Lung CD11b+ cells were isolated from EAE mice 7 DPI and cultured with CD4+ T cells isolated from naïve mouse spleen for 48 h before supernatant harvest and downstream ELISA analysis. Fluorescence-activated cell sorting was used to sort G-MDSCs and M-MDSCs.	• G-MDSCs and M-MDSCs ↑ IL-17A production from CD4+ T cells, promoting Th17 polarization.	
Yi *et al*, 2012, J. Immunol	**G-MDSCs**	**EAE Mice Model** Spleen-derived isolated G-MDSCs cultured with sorted naive CD4+CD25-CD62L+ T cells *ex vivo* in Th17-polarizing media, which contained IL-6, TGF-β, anti-IFN-γ, and IL-4 mAbs.	• G-MDSCs ↑ the expansion of Th17 cells in the presence of IL-6 and TGF-β.	[61]
Radojević *et al*. 2022, Gut Microbes.	**M-MDSCs**	**EAE Mice Model** Bone marrow (BM) cells were differentiated with and without PGE2, producing M-MDSCs-PEG2 and M-MDSCs. Mice received a single dose of M-MDSCs or M-MDSCs-PGE2 a day after EAE induction. Spinal cord and spleen of each mouse collected after sacrifice. *Ex vivo* cell analysis performed with immunofluorescence staining and flow cytometry.	• M-MDSCs-PGE2 administration ↓ Th17 levels in the spinal cord. • M-MDSCs and MDSCs-PGE2 administration ↓ IFN-γ-producing NK cells both in the spinal cord and spleen. • M-MDSCs and MDSCs-PGE2 administration ↑ regulatory T cells population both in the spinal cord and spleen. • M-MDSCs-PGE2 migrated into gut lymphoid tissues and prevented EAE-induced changes in the intestinal barrier markers.	[62]
Parekh *et al*. 2013, J. Immunol	**M-MDSCs, G-MDSCs**	**EAE Mice Model** Mice received α-GalCer or vehicle injection at 0, 4 and 7 DPI. Spinal cord and spleen of each mouse collected after sacrifice. *Ex vivo* cell analysis performed with immunofluorescence staining and flow cytometry.	• M-MDSCs ↑↑ in the spleen and CNS of α-GalCer-treated mice compared with vehicle-treated mice. • G-MDSCs ↑ in the spleen and CNS of α-GalCer-treated mice compared with vehicle-treated mice. • CD4+ T cells CNS infiltration ↓ in α-GalCer-treated mice compared with vehicle-treated mice. • M and G-MDSC depletion completely abrogated disease protection conferred by iNKT cell activation.	[63]
Alabanza *et al*. 2013, J. Immunol	**M-MDSCs, G-MDSCs**	**EAE Mice Model** EAE mice received mAb anti-PC or vehicle i.p. injection on 0, 2, 4, and 6 DPI. CNS and spleen of each mouse collected after sacrifice. *Ex vivo* cell analysis performed with immunohistochemistry and flow cytometry.	• M and G-MDSCs levels ↑ in the spleen of anti-PC mice compared with vehicle-treated mice. • Activated PC binds directly to M and G-MDSCs through PAR-1. • Anti-PC treatment attenuated EAE.	[64]
Moliné‐Velázquez *et al*. 2014, Neurobiol Dis.	**M-MDSCs, G-MDSCs**	**EAE Mice Model** Mice received daily one dose of AM80 in a 0.5% CMC solution administered by oral gavage or CMC solution alone for 5 or 14 days. Spinal cord and spleen of each mouse collected after sacrifice. *Ex vivo* cell analysis performed with immunohistochemistry and flow cytometry.	• M-MDSCs ↓ in the spleen of AM80 treated mice compared with CMC treated mice. • G-MDSCs ↑ in the spleen of AM80 treated mice compared with CMC treated mice. • AM80 ↑ apoptotic M-MDSCs in the spinal cord of EAE mice. • AM80 ↓ apoptotic T cells in the spinal cord of EAE mice.	[65]
King *et al*. 2009, Blood	**M-MDSCs**	**EAE Mice Model** CNS mononuclear cells and peripheral blood cells of each mouse collected after sacrifice and analyzed with flow cytometry.	• M-MDSCs circulating levels and CNS infiltration ↑ before and during EAE exacerbations. • M-MDSCs MHC class II + in the CNS expressed ↑ mRNA encoding p40 (the common subunit of IL-12 and IL-23), p19 (the IL-23–specific subunit), and IL-6, exhibiting a pro-inflammatory phenotype.	[66]
Zhang *et al*. 2022, Pharm. Biol.	**MDSCs, G-MDSCs**	MDSCs isolated from mice were cultured in the presence of PLY, PBS or JAK-1 inhibitor solcitinib (positive control) for 48 h. Specific M-MDSCs surface markers were examined using flow cytometry.	• Number and % of M-MDSCs ↓ after PLY treatment. • % of G-MDSCs ↑ after PLY treatment (but not the absolute number). • PLY inhibited MDSCs expansion and differentiation into M-MDSCs	[67]
		**EAE Mice Model** EAE mice were treated with PLY, PBS or 1 mg/kg FK-506 by i.p. injections for 21 days. Spinal cord and peripheral blood of each mouse collected after sacrifice. *Ex vivo* cell analysis performed with immunofluorescent staining and flow cytometry.	• PLY ↓ M-MDSCs levels in peripheral blood (but not G-MDSCs). • PLY alleviated EAE progression. • + Correlation between MDSCs infiltration and IL-17A levels in the spinal cord of EAE mice was found.	
Hertzenberg *et al*. 2013, Eur J Immunol.	**M-MDSCs**	**EAE Mice Model** EAE was induced in mice at the age of 2 week or 8 weeks. Splenocytes from 2- or 8-week-old mice were collected after sacrifice and evaluated for the frequency of leukocyte subsets with flow cytometry.	• None of the 2-week-old mice showed any clinical signs of EAE, whereas 8/8 mice at the age of 8 weeks developed the condition. • M-MDSCs and plasmacytoid dendritic cells are ↑ in the spleen of 2-week-old mice. • M-MDSCs from the spleen of 2-week-old mice express ↓ MHC class II and ↓ co-stimulatory CD40. • Splenocytes from 2-week-old mice produced ↓ TNF, IL-23, IL-6 and IL-12 and ↑ IL-10 compared to splenocytes from 8-week-old mice.	[69]
Melero-Jerez *et al*. 2019, Neurobiol Dis.	**M-MDSCs, G-MDSCs**	**EAE Mice Model** At the onset of the symptoms EAE mice received a single i.p. injection of 10,000 units recombinant murine IFN-β. Spinal cord and spleen of each mouse collected after sacrifice. *Ex vivo* cells used for *in vitro* cultivation and analyzed with immunofluorescence staining and flow cytometry.	• M-MDSCs ↑ in the spleen of EAE mice after a single injection of IFN-β (no significative change detected in G-MDSCs levels). • Arg-I+ MDSCs ↑ in the CNS of EAE mice after a single injection of IFN-β. • M-MDSCs ↑ T cell suppressive activity after IFN-β treatment *in vivo* and *in vitro*. • IFN-β maintains M-MDSCs undifferentiated state.	[70]
Tanwar 2020, J Autoimmun.	**M-MDSCs, G-MDSCs**	**EAE Mice Model** EAE was induced in WT and IFNAR deficient mice models. CNS of each mouse collected after sacrifice. *Ex vivo* cell analysis performed with flow cytometry, ELISA, and chemokine arrays.	• IFNAR deficient mice develop enhanced EAE. • CD4+Foxp3− T cells from IFNAR deficient mice had an altered capacity to produce multiple chemokines as compared to WT mice. • IFNAR deficient mice showed ↓ in the migration of M-MDSCs and G-MDSCs to the draining lymph nodes during early EAE.	[71]
van der Touw *et al*. 2018, J Immunol.	**M-MDSCs**	**MCA26 Tumor-bearing BALB/c Mice** Tumor-bearing mice model was used to generate murine MDSC *in vivo* and *in vitro*. Suppressive activity of MDSC was assessed in a peptide-mediated proliferation assay of TCR transgenic T cells. Cytokines were measured in culture supernatants with ELISA kits. GA treated M-MDSCs were analyzed with immunoprecipitation and immunoblot.	• GA ↓ proinflammatory responses on M-MDSC through binding to paired Ig-like receptor B, thus ↓ STAT1/NF-kβ pathways and ↑ IL-10 and TGF-β release. • GA ↑ M-MDSC-induced T-reg expansion. • Both GA and M-MDSCs ↓ IL-17 levels.	[72]
Knier *et al*. 2018, Nat Immunol.	**G-MDSCs**	**EAE Mice Model** Mice treated with Ly6G antibody (to deplete G-MDSCs) or rat IgG2a control antibody i.p. every other day, starting 12 DPI. Spinal cord and spleen of each mouse collected after sacrifice. *Ex vivo* cell analysis performed with immunofluorescence staining and flow cytometry. Analysis of cytokine and chemokine levels in murine CSF performed with immunoassay and flow cytometry.	• G-MDSCs ↑ at disease onset and form a persisting population during the recovery phase. • CD19+ B cells ↑ in the CNS of G-MDSCs-depleted mice compared to control. • Co-culture of G-MDSCs isolated from the CNS of WT mice during EAE recovery (21 DPI) with naïve stimulated B cells ↓↓ B cell proliferation. • G-MDSCs depleted mice didn’t recover from clinical signs of disease.	[73]
Camacho-Toledano *et al*. 2022	**M-MDSCs**	**EAE Mice Model** EAE mice treated with FTY720 or vehicle alone every day for 14 days and sacrificed 15 DPI. CNS and spleen samples collected after sacrifice. *Ex vivo* tissue and cells analysis performed with immunohistochemistry, immunostaining, and flow cytometry.	• EAE-FTY720 non-responder mice had ↓↓ M-MDSCs within the whole blood cells compared to EAE-FTY720 responders, prior to treatment initiation. • M-MDSCs had excellent discriminatory power to assess the risk of being responder or non-responder, based on the median value of the maximum peak clinical score, the percentage recovery of the clinical score and the residual clinical score.	[74]

**Abbreviations:** 2-AG, 2-Arachidonoylglycerol; α-GalCer, α-Galactosylceramide; AM80, retinoic acid receptor α (RARα) agonist; Arg-1, arginase 1; CFA, Freund's Complete Adjuvant; CMC, carboxymethylcellulose; CNS, central nervous system; DBI, days before immunization; dLNs, draining lymph nodes; DPI, days post immunization; EAE, experimental autoimmune encephalomyelitis; EDI, escalating dose immunotherapy; ELISA, enzyme-linked immunosorbent assay; FK-506, Tacrolimus; FTY720, Fingolimod; G-MDSCs, granulocytic myeloid-derived suppressor cells; GA, Glatiramer Acetate; i.d., intradermal; i.p., intraperitoneal; IFNAR, Interferon-alpha/beta receptor; IFN-β, Interferon-β; IFN-γ, Interferon-γ; iNKT, invariant Naturak Killer T cells; LN, lymph nodes; M-MDSCs, monocytic myeloid-derived suppressor cells; mAb, monoclonal Antibody; MBP_Ac1-9_4K, synthetic analogue of the acetylated nine‐residue peptide (Ac1-9) of myelin basic protein; MBP_Ac1-9_4Y, synthetic analogue of the acetylated nine‐residue peptide (Ac1-9) of myelin basic protein; MOG, Myelin Oligodendrocyte Glycoprotein; OPCs, oligodendrocyte progenitor cells; PBS, phosphate buffered saline; PC, Protein C; PD-1, Programmed cell Death protein-1; PDL-1, Programmed Death Ligand-1; PLY, Pseudolycorine chloride; RB6-8C5, Ly-6G/Ly-6C Monoclonal Antibody; RT-PCR, Real Time Polymerase Chain Reaction; s.c., subcutaneous; SA–IL-4, serum albumin/IL-4 fusion protein; TCR-transgenic mice, Tg4 T‐cell receptor‐transgenic mouse with a Vα4 Vβ8.2 transgenic TCR specific for the acetylated nine‐residue peptide (Ac1‐9) of MBP; TGF-β, Transforming Growth Factor-β; Th1, T helper type 1; TMEV-IDD, Theiler’s Murine Encephalomyelitis Virus-Induced Demyelinating Disease; UCM-03025, reversible inhibitor of 2-AG degradation; WT, Wild Type.

**Table 2:** Multiple sclerosis and MDSCs.

**Authors, Year, Journal**	**Investigated Cell Type(s)**	**Study Design**	**Summarized Findings**	**References**
D’Amico *et al*. 2022, Front Immunol.	**M-MDSCs**	Demographic/clinical data and peripheral bloods were collected from 52 naive patients recently diagnosed with RRMS and sex/age-matched HCs in a 2:1 ratio. Flow cytometry performed on isolated peripheral blood mononuclear cells to assess immune cell subsets differences between RMMS patients and HCs.	• ↑ M-MDSCs CD14+/HLADR−/^low^ and ↑ CD14+/ CD16+ inflammatory monocytes in RRMS patients compared to HCs.	[75]
Iacobaeus *et al*. 2018, Immunol Cell Biol.	**M-MDSCs, G-MDSCs**	Demographic/clinical data and peripheral bloods were collected from 11 RRMS-relapse, 19 RRMS-remission, 19 SPMS and 20 HCs. Flow cytometry performed on isolated peripheral blood mononuclear cells to assess immune cell subsets differences between MS patients and HCs. Stimulated CD3+ T cells isolated from every subgroup co-cultured in the presence/absence of autologous M-MDSCs.	• ↑ M-MDSCs CD14+/HLADR−/^low^ and ↑ G-MDSCs CD33+/CD15+/CD11b+/HLA-DR^low^ in RRMS-relapse compared with RRMS-remission and SPMS. • ↓ M-MDSCs CD14+/HLADR−/low and ↓ G-MDSCs CD33+/CD15+/CD11b+/HLA-DR^low^ in SPMS compared with HCs. • M-MDSCs in RRMS-relapse, RRMS-remission, and HCs ↓ autologous T cells proliferation compared to their respective controls. • M-MDSCs in SPMS ↑ autologous T cells proliferation compared to their respective controls.	[76]
Cantoni *et al*. 2017, Acta Neuropat.	**M-MDSCs**	Demographic/clinical data and peripheral bloods were collected from 24 naïve RRMS, 10 GA-treated RRMS and 16 HCs. Flow cytometry performed on isolated peripheral blood mononuclear cells to assess immune cell subsets differences between MS patients and HCs. Autologous CFSE-labeled CD4+ T cells were co-cultured (1:2 ratio) with MDSCs isolated from the blood of a control subject in the presence of anti-CD3 and anti-CD28. T cell proliferation in the absence or presence of MDSCs was examined by CSFE dilution and flow cytometry. miRNA-223 expression was assessed in MDSCs isolated from untreated RRMS patients (n = 11) and healthy controls (n = 10) by qPCR.	• M-MDSCs CD14+/HLADR− are ↓ in MS patients compared with HCs. • M-MDSCs in HCs ↓ autologous T cells proliferation. • ↑ miRNA-223 in MS patients compared to HCs. • M-MDSCs suppressive function increased after glucocorticoid treatment in MS patients.	[77]
Ortega *et al*. 2023, Acta Neuropat.	**M-MDSCs**	Post-mortem MS cortical snap-frozen tissue (n=20 SPMS; n=13 PPMS) and six HCs were analyzed. Immunohistochemistry or immunofluorescence staining was performed. Quantification of M-MDSCs was then performed with a confocal microscope.	• M-MDSCs are present in the white matter lesions of MS patients. • M-MDSCs ↑ in AL and rAIL. • M-MDSCs ↓ in cAIL and IL. • M-MDSCs ↑ in high inflammatory areas of both PPMS and SPMS. • M-MDSCs ↓ in PPMS patients with shorter disease duration.	[58]
		Demographic/clinical data and peripheral bloods were collected from 47 MS patients and 26 HCs. Flow cytometry performed on isolated peripheral blood mononuclear cells to assess immune cell subsets differences between MS patients and HCs.	↑ M-MDSC content in MS patients close to relapse associated with ↓ Disability at the time of sampling and ↑ recovery after 12 months of follow-up.	

**Abbreviations:** AL, Active Lesions; cAIL, Active/Inactive Lesions with hypocellular lesion center; CFSE, carboxyfluorescein diacetate succinimidyl ester; GA, Glatiramer Acetate; G-MDSCs, granulocytic myeloid-derived suppressor cells; HCs, healthy controls; IL, Inactive Lesions; miRNA, microRNA; M-MDSCs, monocytic myeloid-derived suppressor cells; MS, Multiple Sclerosis; PPMS, primary progressive Multiple Sclerosis; qPCR, quantitative Polymerase Chain Reaction; rAIL, Active/Inactive Lesions with a rim; RRMS, relapsing-remitting Multiple Sclerosis; SPMS, secondary progressive Multiple Sclerosis.

## LIST OF ABBREVIATIONS

2-AG: 2-arachidonoylglycerol
ADCC: FC-receptor-mediated Antibody-dependent cell-mediated Cytotoxicity
APC: Antigen Presenting Cells
Arg-I: Arginase-I
CBD: Cannabidiol
CD40L: CD40 Ligand
CD62L: L-selectin
CNS: Central Nervous System
CTLA-4: Cytotoxic T Lymphocyte-associated Antigen 4
DCs: Dendritic Cells
DMTs: MHC, CD86, Lys6G, CD11b, Disease-modifying Therapies
FAS-L: Fas Ligand
G-MDSCs: Granulocytic MDSCs
GA: Glatiramer Acetate
HCs: Healthy Controls
IFN-β: Interferon-beta
iNOS: Inducible Nitric Oxide Synthase
M-MDSCs: Monocytic MDSCs
MDSCs: Myeloid-derived Suppressor Cells
MHC: Major Histocompatibility Complex
MPPT: Methylprednisolone Pulsed Therapy
MS: Multiple Sclerosis
NADPH: Nicotinamide Adenine Dinucleotide Phosphate Oxidase
NK: Natural Killer
NO: Nitric Oxide
OPCs: Oligodendrocyte Precursor Cells
PD-L1: Programmed Death-ligand 1
PD-L1: Programmed Death-Ligand 1
PGE2: Prostaglandin E2
PMNs: Polymorphonuclear Cells
PP: Primary Progressive
ROS: Reactive Oxygen Species
RR: Relapsing Remitting
SP: Secondary Progressive
T-reg: T-regulatory
TACE: Tumor Necrosis Factor-α Converting Enzyme
TCR: T-cells Receptor
TGF-β: Transforming Growth Factor Beta
TMEV-IDD: Theiler’s Virus Induced Demyelination Disease
Treg: Regulatory T

## APPENDIX

### (1A) Search Terms Query Box

"Multiple Sclerosis"[All Fields]) AND ("myeloid derived suppressor cells"[MeSH Terms] OR ("myeloid derived"[All Fields] AND "suppressor"[All Fields] AND "cells"[All Fields]) OR "myeloid derived suppressor sc"[All Fields]) AND "Experimental Autoimmune Encephalomyelitis"[All Fields] OR "Thieler's Murine Encephalomyelitis Virus-Induced DemyelinatingDisease" [All Fields].
